# The HEART score has less utility with high sensitivity troponin

**DOI:** 10.3389/fcvm.2026.1867018

**Published:** 2026-07-16

**Authors:** Austin Harris, Kory Heier, Jeffrey F. Spindel, Weston McCowan, Emily Slade, Mansi Parekh, Dealla Samadi, Vedant Gupta

**Affiliations:** 1 Division of Cardiovascular Medicine, Gill Heart and Vascular Institute, University of Kentucky, Lexington, KY, United States; 2 Department of Biostatistics, University of Kentucky, Lexington, KY, United States; 3 Division of General Internal Medicine, University of Kentucky, Lexington, KY, United States; 4 Division of Internal Medicine and Pediatrics, University of Kentucky, Lexington, KY, United States

**Keywords:** acute myocadial infarction, angina, chest pain, coronary artery disease, major adverse cardiovascular events

## Abstract

**Background:**

Clinical risk scores for acute chest pain evaluation were largely validated using older troponin assays. We sought to analyze the HEART score and clinical decision pathways utilizing high sensitivity troponin.

**Methods:**

This was a retrospective study of all adult patients presenting with nontraumatic chest pain to a single center, quaternary-care emergency department over a 6-month time period. Patients were risk-stratified by the HEART score using an institution-specific clinical decision pathway that aligns with current guidelines. Predictive models and optimal cutoff values were constructed via logistic regression for the primary outcome of acute coronary syndrome at presentation or major adverse cardiovascular events within 30 days and additional analysis was performed among risk groups assessing associations of HEART score with negative troponin and electrocardiogram compared to positive troponin or electrocardiogram.

**Results:**

Data from 1,014 patients were included for analysis with 46 patients (4.5%) meeting the primary outcome. The model most associated with the primary outcome utilized electrocardiogram and high-sensitivity troponin only (C-statistic = 0.883), which reliably predicted the absence of the primary outcome in intermediate risk patients. The addition of medical history, risk factors, and age to this model (via the continuous HEART score) did not increase its value (C-Statistic 0.876).

**Conclusion:**

Our study suggests that high-sensitivity troponin and electrocardiogram alone can be used to risk stratify most patients with acute chest pain appropriately. Traditional scoring systems developed with contemporary cardiac troponin assays have less utility in the era of high sensitivity troponin, especially with patients previously deemed intermediate risk.

## Introduction

Chest pain remains the second most common emergency department (ED) presenting complaint in the United States, accounting for 5.5% of all ED visits or over 7 million visits annually (1). Chest pain accounts for a large expenditure of healthcare resources and significant cost, estimated in 2016 at around $1.5 billion dollars annually (2). However, a low percentage of patients presenting with chest pain are diagnosed with acute coronary syndrome (ACS), necessitating the need for efficient schema for diagnosing and excluding ACS (3). Most recently, the 2021 AHA/ACC Guideline for the Evaluation and Diagnosis of Chest Pain 2021 guideline assigned a class 1 recommendation for the use of clinical decision pathways (CDP) to stratify patients into risk categories to facilitate disposition (4). While various risk stratification pathways have been studied, they were initially validated with older cardiac troponin assays (5–8).

High sensitivity cardiac troponin (hs-cTn) assays are more sensitive than traditional assays (9, 10). A baseline hs-cTn below the limit of quantification (LOQ) effectively rules out ACS, with negative predictive values of greater than 98%, improving diagnostic yield and thereby reducing further testing and admissions (9, 11, 12). Current CDPs combine risk stratification scores with hs-cTn assays and have been further validated in prospective studies and randomized controlled trials (13, 14). One of the most commonly used stratification systems and CDPs, the HEART Score, stratifies based on both objective data and medical history. Traditionally, patients who fell into the low-risk group had a 2.5% risk of major adverse cardiac events (MACE) while the intermediate and high-risk group had a 20.3% and 72.7% rate of MACE, respectively (8, 15, 16).

However, since the inclusion of hs-cTn, studies and CDP have deviated from the original HEART CDP, suggesting that in patients deemed low risk, discharge after a single troponin below the LOQ is safe and cost-effective, and supported in the 2023 European Society of Cardiology (ESC) Guidelines (11, 12, 14, 17–20). While there have been efforts to recalibrate the HEART score utilizing new hs-cTn cutoff values (21), the utility of the HEART score, and similar stratification systems, is uncertain when the readily available objective test has near complete sensitivity and negative predictive values (22).

While ESC, ACC/AHA guidelines, and supporting studies recommend additional testing in intermediate-risk patients (18, 23), this patient subgroup is less clearly defined in CDPs in the era of hs-cTn. Therefore, we sought to assess the risk profile of different risk stratification categories of the HEART Score CDP when utilizing hs-cTn.

## Methods

This was a retrospective study of all patients presenting with nontraumatic chest pain to the University of Kentucky Chandler Hospital emergency department (ED) between July and December 2021. Patients were excluded if they were <18 years of age or did not undergo CDP-based assessment (had no ECG and troponin value). Electronic medical records were queried for demographics, clinical risk factors, lab values, diagnosis made during admission, and outcomes after discharge including MI, CVA, hospitalization for heart failure, and cardiac death. Data on cardiac testing during and up to 90 days after hospital presentation were collected including coronary CTA, stress testing, and invasive angiography. This study was reviewed and approved by our institutional IRB (#77310).

### Outcomes and predictor variables

The primary outcome was the composite of ACS at presentation, per the Fourth Universal Definition of Myocardial Infarction, or MACE within 30 days, to account for any potential misclassification at initial presentation. Major adverse cardiovascular events included cardiovascular mortality, hospitalization for heart failure, presentation for myocardial infarction or unplanned revascularization. Cardiovascular mortality was defined as death due to a cardiovascular cause. Planned staged revascularization for residual CAD was not included in the primary endpoint, as it was not deemed a failure of the diagnostic pathway. The predictor variables of interest were the HEART score and its components as defined by Six, Backus, and Kelder, and validated in multiple studies (8, 15, 16).

Only the initial ECG, all of which were interpreted by a single physician blinded to the patient outcome, and initial hs-cTn were used for scoring. Roche Elecsys 5th generation high sensitivity cardiac troponin T assay (F. Hoffman-LaRoche AG, Basel, Switzerland) was utilized. Further detail on the HEART Score and its components are presented in the Supplemental Material (S-1), as are the HEART Score CDP and troponin ranges which were previously published (S-2: Supplementary Figure 1) (24).

### Missing data

Missing data was present in some variables; notably, 39.9% were missing information on family history of coronary artery disease, myocardial infarction, or sudden cardiac death in a first degree relative prior to age 65; and 21.1% were missing information on tobacco use. The frequency of missing data for all variables can be found in the supplemental material (S-3: Supplementary Table 1). Missing data in race, clinical diagnoses and history (atherosclerosis, hypertension, HLD, diabetes, subjective history (per HEART score of nonspecific symptoms, moderately or highly suspicious for clinically significant coronary artery disease), obesity, family history of coronary artery disease or sudden cardiac death in a first degree relative prior to age 65, tobacco use) were imputed using multiple imputation by chained equations (25, 26). For each of these variables, the imputation model included all aforementioned variables (less the variable itself), plus age, sex, ECG, troponin, and the primary outcome (ACS at presentation or MACE within 30 days). Regression-based predictive mean matching was used with 50 chain iterations and 20 imputed datasets. Convergence was verified by visual inspection of trace plots. Missing composite scores (e.g., risk factor sum, ‘R’ component of HEART score) were calculated after imputation of the individual components.

### Statistical analysis

Summary statistics were generated for each variable stratified by the primary outcome (ACS at presentation or MACE event within 30 days of visit). Categorical variables were reported using frequencies and column percentages (%). Continuous variables were tested for normality using the Shapiro–Wilk normality test along with histograms. Continuous variables were reported using medians and first/third quartiles [Q1,Q3]. *P*-values for categorical variables were generated using either Pearson's chi-square tests or Fisher's exact tests. *P*-values for continuous variables were given using the Wilcoxon rank sum test.

Using logistic regression, we considered four models for predicting ACS at presentation or MACE within 30 days. Model 1, hs-cTn only, was created to determine the association of a single troponin value with the primary outcome. Current guidelines recommend a single positive troponin rules patients in for additional stratification and assessment (18, 23). The HEART CDP designates patients as low risk with a score ≤3 and an initial hs-cTn < ULN. This model sought to assess the association of initial hs-cTn with outcomes in patients of *all* HEART scores.

Model 2, assessing ECG and hs-cTn as separate variables, was created with the hypothesis that these two variables would best reclassify unstable angina as either noncardiac pain or an ACS event (27). Furthermore, ECG changes occur earlier in the ischemic cascade than troponin elevations, even hs-cTn.

Model 3, using the HEART score as a continuous variable, was created to compare our hypothesis that troponin and ECG alone were adequate in risk stratification compared to the traditional HEART score, and to assess if different cut-off values were appropriate when utilizing hs-cTn.

Results from a large meta-analysis associated elevated baseline hs-cTn levels in the general population, outside an acute care setting, with long-term cardiovascular risk (28). We suspected this finding to be colinear with comorbidities and medical history, so created Model 4 to assess the utility of the HEART score in patients with normal troponin values. Model 4 uses HEART score risk levels (low, intermediate, high) and hs-cTn to assess if history, comorbidities, subjective symptom assessment, and to a degree, chronic ECG changes, were associated with the primary outcome in the absence of positive biomarkers.

C-statistics and 95% confidence intervals from each logistic regression model are reported to assess the predictive power of each model. Additionally, within each HEART score risk level, Fisher's exact tests were used to compare the risk of the primary outcome between those with or without a positive troponin/ECG.

All statistical tests were two-sided and statistical significance was defined as *p*-value ≤0.05. Analysis was performed in R programming language, version 4.1.1 (R Foundation for Statistical Computing, Vienna, Austria).

## Results

In total, 1,346 charts were reviewed. After making exclusions for age <18 (*n* = 99), chest pain due to trauma or lack of CDP assessment (no troponin value and ECG) (*n* = 233), data from 1,014 patients were included for analysis.

Demographic and clinical characteristics are presented in S-3: Supplementary Table 1. Of the 1,014 patients, 52% were female and 69% identified as Caucasian. Our cohort had frequent comorbidity with 21% having known atherosclerotic disease, 43% having hypertension, 20% having diabetes, 43% being obese, and 24% reported using tobacco within the last 3 months. Missing risk factors were mainly family history of coronary artery disease, myocardial infarction or sudden cardiac death in a first degree relative prior to age 65 (39.9% missing), and tobacco use within the last 3 months (21.1% missing).

The primary outcome was met by 46 patients (4.5%). There were 11 patients (1.1%) who were hospitalized for HF within 30 days of ED visit, 3 patients (0.3%) presented with stroke within 30 days of ED visit, 1 patient (0.1%) had MI within 30 days of ED visit, 1 patient (0.1%) had unplanned revascularization within 30 days of ED visit, and 32 patients (3.2%) presented with ACS at the index ED visit.

### HEART score models

Logistic regression results for each of the four models are presented in Table 1. In Model 1, troponin value alone was highly associated with the primary outcome (C-Statistic 0.862). A troponin value >1x gender specific cutoff had an odds ratio of ACS at presentation/MACE within 30 days of 11.3 compared to troponin <1x cutoff. Initial hs-cTn ≥52 ng/L had an odds ratio of 70.7 for ACS at presentation/MACE within 30 days.

**Table 1:** Associations with heart events and predictive ability of each model.

Model	Odds ratio (95% CI)	C-statistic (95% CI)
Model 1: Troponin only		0.862 (0.860, 0.864)
T = 0	Ref	
T = 1	11.3 (4.6, 27.9)	
T = 2	70.7 (29.8, 167.9)	
Model 2: ECG + Troponin		0.883 (0.881, 0.885)
ECG		
E = 0	Ref	
E = 1	0.2 (0.0, 0.7)	
E = 2	7.3 (2.8, 19.4)	
Troponin		
T = 0	Ref	
T = 1	12.3 (4.8, 31.6)	
T = 2	65.1 (26.1, 162.4)	
Model 3: HEART score (continuous)	2.2 (1.9, 2.7)	0.876 (0.870, 0.883)
Model 4: HEART score risk level + Troponin		0.875 (0.874, 0.876)
Low HEART Score (<=3) and T = 0	Ref	
Mid HEART Score (4–6) and T = 0	4.1 (0.8, 20.4)	
High HEART Score (7+) and T = 0	435.5 (30.5, 6214.2)	
T = 1 or 2	54.1 (16.5, 177.8)	

In Model 2, inclusion of the troponin value and ECG score as separate variables also provided high predictive power for the primary outcome (C-Statistic of 0.883). The odds of ACS/MACE among those with E = 1 are 80% lower than among those with E = 0, after adjusting for T. The odds of ACS/MACE among those with E = 2 are 7.3 times higher than among those with E = 0, after adjusting for T. The odds of ACS/MACE among those with T = 1 and T = 2 are 12.3 and 65.1 times higher, respectively, than among those with T = 0, after adjusting for E.

In Model 3, the HEART score itself, treated as a continuous variable, was highly associated with the primary outcome (C-Statistic 0.876). Each 1-point increase in the HEART score was associated with a 2.2 times higher odds of ACS/MACE at 30 days, regardless of which component of the HEART score was responsible for the point.

Model 4 considered four groups: three traditional levels of HEART score risk (low: 0–3, moderate: 4–6, high: 7+) with negative troponin as well as positive troponin (T > 0). This categorization of risk was also highly associated with the primary outcome (C-Statistic 0.875). In this model, amongst those with negative troponin value, a HEART score of 4–6 was associated with mildly elevated risk for ACS/MACE compared to a HEART score of 0–3 (OR = 4.1). Amongst those with negative troponin value, a HEART score of >7 was highly associated with the primary outcome, though the confidence interval was limited by low incidence, and HEART >7 with a negative troponin by design must have an abnormal ECG. Compared to a HEART score <3 with negative troponin, any elevation in troponin value, regardless of HEART score, was associated with a 54.1 times higher odds ratio of ACS at presentation or MACE within 30 days.

### Optimal cutoff values

Observed incidence of heart events based on the optimal cutoff values for each model are presented in Table 2. The best discriminatory ability is achieved utilizing a combination of ECG and troponin (Model 2), with a troponin score of 2 (>3x ULN), a negative troponin with ECG score of 2, or a combination of ECG and troponin abnormalities predicting occurrence of the primary outcome. A troponin score of >0 (greater than 1x upper limit of normal) alone had a similar area under the curve (Model 1). Using the continuous HEART score (Model 3), a score ≥ 4 presented the optimal cutoff value.

**Table 2:** Optimal cut-off values for predicting ACS at presentation or MACE within 30 days.

Model	Model prediction (0 = no heart event, 1 = heart event)	Observed incidence of heart event
Model 1: Troponin only		
T = 0	0	1.0%
T > 0	1	19.9%
Model 2: ECG + Troponin		
(E = 0, T = 0), (E = 1, T = 0), (E = 1, T = 1)	0	0.8%
(E = 2, T = 0), (E = 0, T = 1), (E = 2, T = 1), (E = 0, T = 2), (E = 1, T = 2), (E = 2, T = 2)	1	23.9%
Model 3: HEART score (continuous)		
< 4	0	0.6%
>=4	1	12.4%
Model 4: HEART score risk level + Troponin		
Low HEART score or Mid HEART score	0	0.7%
High HEART score or T > 0	1	20.6%

### Effects of troponin and ECG contribution to risk groups

**Figure 1:**
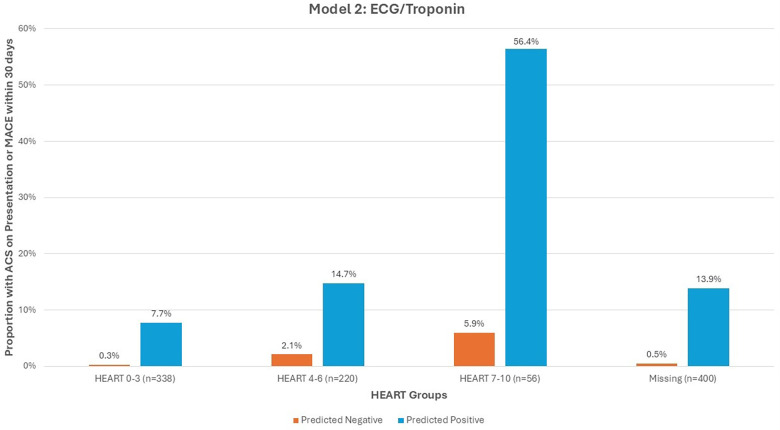
Proportion of patients with MACE/ACS by HEART score and ECG/troponin status.

Figure 1 (and S-4: Supplementary Table 2) illustrates the concordance between observed patient outcomes and predicted outcomes from the best performing model (Model 2), stratified by HEART score risk group (0–3, 4–6, 7–10, missing). Specifically, it displays the proportion of patients who experienced ACS at presentation or MACE within 30 days among those who were predicted to experience the outcome (i.e., those with positive troponin and/or ECG) and among those who were predicted not to experience the outcome. Rather than a dichotomous negative troponin and negative ECG (E = 0 & T = 0) representing the group predicted not to meet the outcome, those with E = 1 & T = 0 or E = 1 & T = 1 were also predicted not to meet the outcome by Model 2. Of the 851 patients predicted not to experience the primary outcome, there were 44 patients meeting E = 1 & T = 1 criteria and 77 patients meeting E = 1 & T = 0 criteria.

Having positive troponin and/or ECG (i.e., positive prediction in Model 2) was associated with higher observed incidence of ACS/MACE amongst those with HEART score 4–6, HEART score 7–10, or missing components of HEART score (*p* < 0.001, S-4: Supplementary Table 2). Among those with HEART score 0–3, the observed incidence of ACS/MACE was 25 times higher among those predicted to have ACS/MACE (7.7%) than among those not predicted to have ACS/MACE (0.3%), though this result was not statistically significant (*p* = 0.076) due to the small number of people predicted to have ACS/MACE in this group.

Furthermore, the one patient in the low risk group who met the primary outcome but was predicted not to had an event that was likely unrelated, presenting 2 weeks after initial evaluation due to acute heart failure from stress cardiomyopathy and had coronary angiography revealing no atherosclerotic disease.

In the moderate risk (HEART score 4–6) group, the three who met the primary outcome but were predicted not to by Model 2 had history as follows: 1) a 55-year-old male with chronic heart failure with reduced ejection fraction and obesity with BMI 64.5, nonadherent with medications, ruled out for ACS at index presentation and re-presented 1 day later with chest pain and troponin trend of 16 to 26 ng/L, managed medically for NSTEMI with inability to obtain perfusion study or coronary CTA due body habitus, 2) a 48-year-old male with history of 2v CABG and mechanical aortic valve replacement was ruled out in the emergency department for ACS and was admitted 25 days later with acute CVA after warfarin nonadherence, 3) a 51-year-old male with history of multiple PCIs, was ruled out in the emergency department for ACS and re-presented 9 days later with NSTEMI due to in-stent-restenosis requiring PCI.

## Discussion

Our study sought to further clarify the utility of the HEART score and CDP in the era of high sensitivity troponin assays. The primary findings in this investigation are 1) when using high sensitivity troponin, there was not a meaningful difference in performance between just ECG and troponin and the full HEART score, and 2) in patients with negative troponin, event rate is low, regardless of HEART score.

Our results suggest that hs-cTn and ECG alone were adequate in risk stratification for ACS at presentation or MACE within 30 days. Compared to ECG and hs-cTn (Model 2) alone, the addition of history, risk factors, and age via the continuous HEART score (Model 3) did not improve the ability to discriminate between those with/without the primary outcome. A hs-cTn greater than gender specific ULN or a high risk HEART Score, which by definition must have an abnormal ECG or troponin, were consistently stratified as high risk by the optimal cutoff values in all models. A significantly positive hs-cTn (T = 2) and/or ECG (E = 2) predicted the primary outcome in the entire cohort, regardless of HEART Score or missing components (*p* < 0.001).

There was an unanticipated, perhaps spurious, finding in our models. Interestingly, when considering ECG and troponin in Model 2, an ECG score of 1 (LBBB, complete RBBB, pacemaker rhythm, LVH criteria) had an 80% reduction in odds of the primary outcome (OR = 0.2) compared to those that had an ECG value of 0 after adjusting for troponin. An ECG score of 2 (ST segment depression), regardless of troponin value was highly associated with the primary outcome. Furthermore, this model (Model 2) had the highest overall C-statistic. We hypothesize that this is explained by bundle branch blocks, LVH criteria, and pacemaker rhythms accounting more for historical than acute changes or short-term risk. One caveat is that our study design did not allow us to compare these abnormal ECGs to each patient's previous ECGs and assess for changes.

Since high sensitivity troponin assays detect troponin at levels 10 to 100 times lower than previous assays, sensitivity and negative predictive value are extremely high (9, 22). Therefore, risk stratification systems and CDP developed with contemporary troponin assays may over-assess patients by relying equally on long-term risk factors. Likewise, Stopyra et al. found that use of the HEART CDP resulted in over-testing low-risk patients (29).

Compared to the HEART Score, other stratification scores are similarly impaired when combined with hs-cTn, specifically the TIMI scoring system was developed with troponin I, and CDPs utilizing TIMI scores (such as ADAPT), have not been reassessed with hs-cTn (5, 7). At odds with these is the EDACS score which was developed and validated without using troponin, though the subsequent CDP does include negative troponin as a decision point. Based largely on symptom character, this scoring system and CDP may best be suited for stratification in the era of hs-cTn and was shown to be superior in identifying low risk patients when compared to ADAPT (6, 13).

### Low-risk patients (HEART 0–3)

For low-risk patients (HEART score 0–3), the models and optimal cutoff values for predicting ACS/MACE in our study align with current guidelines (18, 23). A positive troponin value in Model 1 was the optimal cutoff value for predicting ACS/MACE, regardless of risk category, and would prompt a second troponin lab for measurement of delta by the CDP. When considering the continuous HEART score (Model 3), a score <4 was defined as the optimal cutoff for rule out of ACS, the same values currently accepted in the HEART CDP for accelerated rule out of ACS and allowing for discharge after a single troponin less than the LOQ.

### Intermediate-risk patients (HEART 4–6)

The disposition of intermediate-risk patients has varied significantly over the past few decades (27). When initially developed, risk stratification with the HEART score suggested hospital admission for patients with greater than low risk due to higher incidence of ACS diagnosis and the time needed to differentiate stable and unstable angina (UA) from myocardial infarction with less sensitive troponin assays (8). With the implementation of hs-cTn, diagnoses of UA became less frequent (30, 31). Furthermore, all-cause mortality at 1 year in patients diagnosed with UA by hs-cTn are similar to patients in the general population or patients with non-cardiac chest pain, though rates of non-fatal MI at 1 year remain higher (32, 33). Therefore, the observation period and 6–12 h protocols for troponin trending are no longer necessary, and focus has been shifted to additional noninvasive testing for stratification and diagnosis (18, 23).

In our cohort, Model 3 identified HEART Score ≥4 as the optimal cut-off for predicting the primary outcome. The event rate associated with this cutoff value was 12.4% vs. 0.6% for HEART <4. Compared to hs-cTn only (Model 1), the continuous HEART Score (Model 3) had a higher C-Statistic but lower percentage of observed incidence above the predictive cut-off value. The continuous HEART score also had a lower C-statistic and observed incidence among those above the predictive cut-off value compared to hs-cTn and ECG (Model 2). In Model 4, the odds of the primary outcome for those with HEART 4–6 with a negative troponin was 4.1 times higher than the odds of the primary outcome for those with HEART <4 with a negative troponin. However, a troponin value higher than ULN regardless of HEART Score was significantly higher (OR 54.1) than HEART <4 with a negative troponin. The observed incidence of the primary outcome was 0.7% for HEART <7 with a negative hs-cTn. Because of this, the optimal cutoff value in Model 4 (C-Statistic 0.875) was HEART ≥7 *or* a hs-cTn > ULN.

In intermediate-risk patients, the incidence of ACS/MACE was significantly lower among those with a negative troponin and ECG or mildly abnormal findings compared to those with either a significantly positive troponin and/or ECG (E = 2) (*p* < 0.001). This direct comparison shows that the key drivers of outcomes in patients presenting with chest pain and an intermediate-risk HEART score may be the hs-cTn and ECG specifically. Supporting this is a comparison of Model 2 and Model 3. The addition of history, age, and risk factors via the continuous HEART score did not improve the c-statistic with ACS/MACE at 30 days compared to ECG and hs-cTn alone.

The ACC/AHA guidelines state that patients with acute chest pain and a risk of 30 day MACE <1% should be classified as low risk (23). In our cohort, intermediate risk patients with a negative troponin and ECG had a 30 day ACS/MACE event rate above this threshold (2%), though this encompassed 3 events, 2 with multiple confounders, as described in the results section. Our results suggest that stratification with ECG and troponin alone may select appropriate patients for additional testing in the acute setting and re-classify others as low risk.

### High-risk patients (HEART 7–10)

Per the HEART Score, patients identified as high risk must by design have an abnormal ECG and/or troponin value. This is reflected in our results by patients with a HEART score ≥7 and negative initial hs-cTn (Model 4) having odds of ACS/MACE at 30 days that is 435 times higher than those with HEART score ≤3, though this result should be interpreted with caution as there were few patients in this group. In all models, patients considered high risk by the HEART score met criteria predicting an ACS/MACE event, except in the specific instance of HEART = 7 with either a bundle branch block, paced rhythm, or LVH criteria on ECG and a negative hs-cTn (Model 2).

## Limitations and conclusion

An important limiting factor in our study was that approximately half of the participants were missing information on at least one risk factor, mostly smoking history and family history. To address this limitation, we decided the best approach was to impute missing data. To address this limitation, we used multiple imputation to impute the missing data. While multiple imputation is a robust and widely accepted method used to account for uncertainty in the missing data, it still relies on assumptions about the missingness mechanism and may introduce bias if those assumptions are violated. Our study had several other limitations. We did not consider second troponin values if they were drawn, therefore no assessment of troponin delta was performed. While this was intentional and meant to focus on initial risk stratification and rule-out, adding delta troponin to our results would increase specificity of stratification and diagnosis.

Also, being a retrospective study, we could not clearly define timeline of symptom onset and initial labwork. Multiple studies and the ACC/AHA guidelines emphasize that rapid rule-out cannot safely be performed if symptom onset was < 3 h at time of initial hs-cTn. However, if a substantial portion of patients had troponin values drawn within 3 h of symptom onset, our results, suggesting that troponin values are the main drivers of disposition, would be only more strongly supported.

In conclusion, our results suggest that for patients presenting to the emergency department with acute, non-traumatic chest pain, ECG and hs-cTn are the variables most associated with ACS at presentation or MACE within 30 days. By the HEART CDP, low risk patients and high-risk patients are easily identified, however, careful consideration of hs-cTn may be able to reclassify intermediate risk patients.

## Data Availability

The raw data supporting the conclusions of this article will be made available by the authors, without undue reservation.
