# Supplementary material for: The HEART score has less utility with high sensitivity troponin
Source: Front Cardiovasc Med. 2026 Jul 16;13:1867018. doi: 10.3389/fcvm.2026.1867018 (PMC13422415; doi:10.3389/fcvm.2026.1867018)
Supplement: Supplementary file 1 [file Supplementaryfile1.docx]

**Supplemental Material**

**S-1) HEART Score Criteria**

The HEART score utilizes 5 criteria, scored 0-2.

**History:** Subjective assessment of history is classified as nonspecific, moderately suspicious for clinically significant coronary artery disease, and highly suspicious.

**Electrocardiogram:** ECG is classified as normal, 1 point is given for presence of a left bundle branch block, complete right bundle branch block, pacemaker rhythm, typical abnormalities of left ventricular hypertrophy, and 2 points are given if there is significant ST depression (>0.5 mm) in the absence of a bundle branch block or LVH. For purposes of our study, we also included ST elevation meeting ischemic criteria, after specifically excluding clear repolarization abnormalities and diffuse ST elevation with PR depression consistent with pericarditis. ECG’s were retrospectively reviewed by a single observer who was blinded to the medical and encounter history of the individual patient. Only the first EKG obtained during the encounter was scored.

**Age** is scored as <45 years, 45-64 years, and >65 years.

**Risk** **factors**: Risk factors include diabetes mellitus, hypertension, current smoking within the last 90 days, hypercholesterolemia, family history of coronary artery disease, myocardial infarction, or sudden cardiac death in a first degree relative prior to age 65, obesity (BMI >30 kg/m^2^). Number of risk factors were summated and 0 points were given if the patient had no risk factors, 1 point was given if the patient had 1-2 risk factors, and 2 points were given if the patient had 3 or more risk factors, or a personal history of atherosclerosis (prior myocardial infarction, percutaneous coronary intervention, coronary artery bypass grafting, cerebrovascular accident, transient ischemic attack, or peripheral arterial disease).

**Troponin:** The first high sensitivity troponin (Roche Diagnostics, Indianapolis, IN, USA) drawn at each encounter was scored as 0 points if the value was less than gender-specific cutoff (<14 ng/L for females, <19 ng/L for males), 1 point was given if the hs-cTnT was above the gender specific cutoff and <52 ng/L for males and females, and 2 points were given if the hs-cTnT was greater than 52 ng/L. These cutoff values were defined by initial hs-cTnT value used in published 0/1 hour algorithms as a rule-in for ACS, (Roffi et al.) and were utilized clinically at the time of patient presentation by our institution’s HEART Score CDP.

**S-2) Supplemental Figure 1. Institutional HEART Score clinical decision pathway for patients presenting to the emergency department.**

**
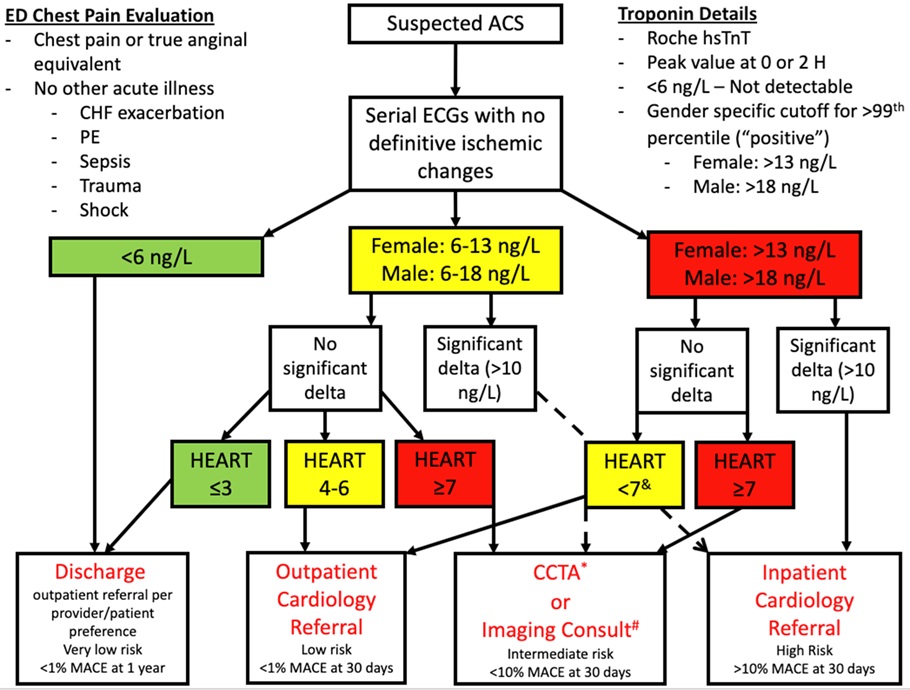
**

**S-3) Table 1 - Demographics and risk factors.**

*History refers to subjective assessment of symptoms for the likelihood of coronary artery disease as the cause (nonspecific, moderately suspicious, or highly suspicious). ^Family history is dichotomous variable of coronary artery disease, myocardial infarction, or sudden cardiac death in a first degree relative prior to age 65. ^#^Risk Factor Sum is the summation of all medical risk factors (diabetes, hypertension, current tobacco smoking, hypercholesterolemia, family history, obesity, and personal history of atherosclerosis defined as prior myocardial infarction, coronary intervention, bypass grafting, cerebrovascular accident, transient ischemic attack, or peripheral arterial disease).

|  | **No Primary Outcome** | **Primary Outcome** | **Overall** |
| --- | --- | --- | --- |
|  | **(N=968)** | **(N=46)** | **(N=1014)** |
| **Age** |  |  |  |
| Median [Q1, Q3] | 45.0 [32.0, 58.0] | 58.5 [49.0, 65.0] | 45.0 [32.0, 59.0] |
| **Sex** |  |  |  |
| Female | 509 (52.6%) | 17 (37.0%) | 526 (51.9%) |
| Male | 459 (47.4%) | 29 (63.0%) | 488 (48.1%) |
| **Race** |  |  |  |
| Asian | 18 (1.8%) | 0 (0%) | 18 (1.8%) |
| Black or African American | 160 (16.8%) | 8 (17.8%) | 168 (16.8%) |
| Hispanic | 80 (8.4%) | 0 (0%) | 80 (8.0%) |
| Native American or Alaskan | 2 (0.2%) | 0 (0%) | 2 (0.2%) |
| Native Hawaiian or Pacific Islander | 2 (0.2%) | 0 (0%) | 2 (0.2%) |
| Other | 7 (0.7%) | 0 (0%) | 7 (0.8%) |
| Unknown | 15 (1.6%) | 1 (2.2%) | 16 (1.7%) |
| White, Non-Hispanic | 669 (70.2%) | 36 (80.0%) | 705 (68.5%) |
| Missing | 15 (1.5%) | 1 (2.2%) | 16 (1.6%) |
| **Atherosclerosis** | |  |  |
| 0 | 772 (80.3%) | 20 (43.5%) | 792 (78.8%) |
| 1 | 191 (19.1%) | 26 (56.5%) | 217 (20.7%) |
| Missing | 5 (0.5%) | 0 (0%) | 5 (0.5%) |
| **Hypertension** | |  |  |
| 0 | 571 (59.0%) | 8 (17.4%) | 579 (57.1%) |
| 1 | 397 (41.0%) | 38 (82.6%) | 435 (42.9%) |
| **HLD** |  |  |  |
| 0 | 743 (76.8%) | 19 (41.3%) | 762 (75.3%) |
| 1 | 220 (22.6%) | 26 (56.5%) | 246 (24.0%) |
| Missing | 5 (0.5%) | 1 (2.2%) | 6 (0.6%) |
| **Diabetes** |  |  |  |
| 0 | 780 (81.0%) | 25 (54.3%) | 805 (79.9%) |
| 1 | 185 (18.5%) | 19 (41.3%) | 204 (19.5%) |
| Missing | 3 (0.3%) | 2 (4.3%) | 5 (0.5%) |
| **History*** |  |  |  |
| 0 | 556 (59.4%) | 9 (19.6%) | 565 (57.7%) |
| 1 | 375 (36.8%) | 10 (21.7%) | 385 (36.2%) |
| 2 | 30 (3.0%) | 27 (58.7%) | 57 (5.4%) |
| Missing | 7 (0.7%) | 0 (0%) | 7 (0.7%) |
| **Obesity** |  |  |  |
| 0 | 538 (56.3%) | 24 (52.2%) | 562 (56.1%) |
| 1 | 422 (42.8%) | 21 (45.7%) | 433 (43.0%) |
| Missing | 8 (0.8%) | 1 (2.2%) | 9 (0.9%) |
| **Family History^** | |  |  |
| 0 | 419 (43.1%) | 21 (45.7%) | 440 (43.2%) |
| 1 | 158 (16.3%) | 11 (23.9%) | 169 (16.6%) |
| Missing | 391 (40.4%) | 14 (30.4%) | 405 (39.9%) |
| **Tobacco** |  |  |  |
| 0 | 525 (53.7%) | 28 (60.9%) | 553 (54.0%) |
| 1 | 234 (24.2%) | 13 (28.3%) | 247 (24.4%) |
| Missing | 209 (21.6%) | 5 (10.9%) | 214 (21.1%) |
| **Risk Factor Sum^#^** | |  |  |
| 0 | 84 (8.6%) | 1 (2.2%) | 85 (8.3%) |
| 1 | 130 (13.4%) | 5 (10.9%) | 135 (13.3%) |
| 2 | 125 (12.4%) | 6 (13.0%) | 131 (12.4%) |
| 3 | 74 (7.7%) | 5 (10.9%) | 79 (7.8%) |
| 4 | 62 (6.3%) | 2 (4.3%) | 64 (6.2%) |
| 5 | 24 (2.4%) | 4 (8.7%) | 28 (2.7%) |
| 6 | 0 (0%) | 2 (4.3%) | 2 (0.2%) |
| Missing | 469 (48.5%) | 21 (45.7%) | 490 (48.3%) |
| **RBBB** |  |  |  |
| 0 | 935 (96.6%) | 45 (97.8%) | 980 (96.6%) |
| 1 | 33 (3.4%) | 1 (2.2%) | 34 (3.4%) |
| **LBBB** |  |  |  |
| 0 | 959 (99.1%) | 46 (100%) | 1,005 (99.1%) |
| 1 | 9 (0.9%) | 0 (0%) | 9 (0.9%) |
| **LVH** |  |  |  |
| 0 | 896 (92.6%) | 45 (97.8%) | 941 (92.8%) |
| 1 | 72 (7.4%) | 1 (2.2%) | 73 (7.2%) |
| **ST Depressions** | |  |  |
| 0 | 945 (97.6%) | 38 (82.6%) | 983 (96.9%) |
| 1 | 23 (2.4%) | 8 (17.4%) | 31 (3.1%) |
| **ST Elevations** | |  |  |
| 0 | 960 (99.2%) | 38 (82.6%) | 998 (98.4%) |
| 1 | 8 (0.8%) | 8 (17.4%) | 16 (1.6%) |
| **ST Early Repolarization** | | |  |
| 0 | 946 (97.7%) | 44 (95.7%) | 990 (97.6%) |
| 1 | 22 (2.3%) | 2 (4.3%) | 24 (2.4%) |
| **H category** |  |  |  |
| 0 | 556 (59.4%) | 9 (19.6%) | 565 (57.7%) |
| 1 | 375 (36.8%) | 10 (21.7%) | 385 (36.2%) |
| 2 | 30 (3.0%) | 27 (58.7%) | 57 (5.4%) |
| Missing | 7 (0.7%) | 0 (0.0%) | 7 (0.7%) |
| **E category** |  |  |  |
| 0 | 815 (84.2%) | 29 (63.0%) | 844 (83.2%) |
| 1 | 131 (13.5%) | 2 (4.3%) | 133 (13.1%) |
| 2 | 22 (2.3%) | 15 (32.6%) | 37 (3.6%) |
| **A Category** |  |  |  |
| 0 | 479 (49.5%) | 8 (17.4%) | 487 (48.0%) |
| 1 | 344 (35.5%) | 25 (54.3%) | 369 (36.4%) |
| 2 | 145 (15.0%) | 13 (28.3%) | 158 (15.6%) |
| **R Category** |  |  |  |
| 0 | 83 (8.5%) | 1 (2.2%) | 84 (8.2%) |
| 1 | 221 (22.5%) | 7 (15.2%) | 228 (22.2%) |
| 2 | 274 (19.6%) | 31 (37.0%) | 305 (20.3%) |
| Missing | 390 (49.3%) | 7 (15.2%) | 397 (39.2%) |
| **T Category** |  |  |  |
| 0 | 815 (79.4%) | 8 (17.4%) | 823 (76.7%) |
| 1 | 117 (11.4%) | 13 (28.3%) | 130 (12.1%) |
| 2 | 36 (3.5%) | 25 (54.3%) | 61 (5.7%) |
| **HEART/Troponin Groups** | | |  |
| Low | 326 (30.1%) | 1 (2.2%) | 327 (28.9%) |
| Mid | 134 (9.4%) | 3 (2.2%) | 137 (9.1%) |
| High | 1 (0%) | 2 (2.2%) | 3 (0.1%) |
| Troponin | 153 (14.9%) | 38 (82.6%) | 191 (17.8%) |
| Missing | 354 (36.6%) | 2 (4.3%) | 356 (35.1%) |

**S-4) Supplemental Table 2. Comparison of event rates in low, intermediate, and high risk HEART scores when stratified by predicted outcome from Model 2.** **Data is depicted in Figure 1.**

|  | **Predicted not to have ACS/MACE**  **(E=0, T=0), (E=1, T=0), (E=1, T=1)** | | **Predicted to have ACS/MACE**  **(E=2, T=0), (E=0, T=1), (E=2, T=1), (E=0, T=2), (E=1, T=2), (E=2, T=2)** | |  |
| --- | --- | --- | --- | --- | --- |
| **HEART Category** | **n** | **Observed incidence of MACE/ACS** | **n** | **Observed incidence of MACE/ACS** | **P-value** |
| HEART Score 0-3 | 325 | 1 | 13 | 1 | 0.076 |
| HEART Score 4-6 | 145 | 3 | 75 | 11 | <0.001 |
| HEART Score 7-10 | 17 | 1 | 39 | 22 | <0.001 |
| Missing Components of HEART Score | 364 | 2 | 36 | 5 | <0.001 |
| **Total** | **851** | **7** | **163** | **39** | **<0.001** |
